# Supplementary material for: Antispasmodic Effect of Alstonia boonei De Wild. and Its Constituents: Ex Vivo and In Silico Approaches
Source: Molecules. 2023 Oct 13;28(20):7069. doi: 10.3390/molecules28207069 (PMC10609272; doi:10.3390/molecules28207069)
Supplement: Supplementary file 1 [file molecules-28-07069-s001.zip › molecules-2593755-supplementary.pdf]

Antispasmodic Effect of *Alstonia boonei* De Wild. and Its Constituents: Ex Vivo and In Silico Approaches

Opeyemi Josephine Akinmurele [1,2,3], Mubo Adeola Sonibare [2,*], Anthony A. Elujoba [4], Akingbolabo Daniel Ogunlakin [5,6,*], Oloruntoba Emmanuel Yeye [6,7], Gideon Ampoma Gyebi [8], Oluwafemi Adeleke Ojo [5] and Abdullah R. Alanzi [9]

[1] Department of Pharmacognosy, Faculty of Pharmacy, Madonna University, Elele 512101, Nigeria; opeyemiakinmurele@gmail.com
[2] Department of Pharmacognosy, Faculty of Pharmacy, University of Ibadan, Ibadan 200005, Nigeria
[3] Comsat International Institute of Technology (CIIT), Abbotabad 22020, Pakistan
[4] Department of Pharmacognosy, Faculty of Pharmacy, Obafemi Awolowo University, Ile-Ife 220101, Nigeria; tonyelu@yahoo.com
[5] Phytomedicine, Molecular Toxicology, and Computational Biochemistry Research Laboratory (PMTCB-RL), Department of Biochemistry, Bowen University, Iwo 232101, Nigeria; oluwafemiadeleke08@gmail.com
[6] H. E. J. Research Institute of Chemistry, International Center for Chemical and Biological Sciences, University of Karachi, Karachi 75270, Pakistan; bishopemmy2010@yahoo.com
[7] Department of Chemistry, Faculty of Science, University of Ibadan, Ibadan 200005, Nigeria
[8] Natural products and Structural (Bio-Chem)-Informatics Research Laboratory (NpsBC-RI), Department of Biochemistry, Bingham University, Karu *961105*, Nigeria; gideonagyebi@gmail.com
[9] Department of Pharmacognosy, College of Pharmacy, King Saud University, *Riyadh 12271*, Saudi Arabia; aralonazi@ksu.edu.sa
* Correspondence: sonibaredeola@gmail.com (M.A.S.); gbolaogunlakin@gmail.com (A.D.O.); Tel.: +234-7037883049 (A.D.O.)

Supplementary Data

Table S1. Binding site coordinates of target proteins.

Dimensions	human PPARgamma-LBD (Å)	human carbonic anhydrase isozyme I (Å)
center_x	13.209	13.361
center_y	48.058	36.104
center_z	61.550	15.657
Size x	19.518	19.518
Size y	18.094	18.094
Size z	18.281	18.281

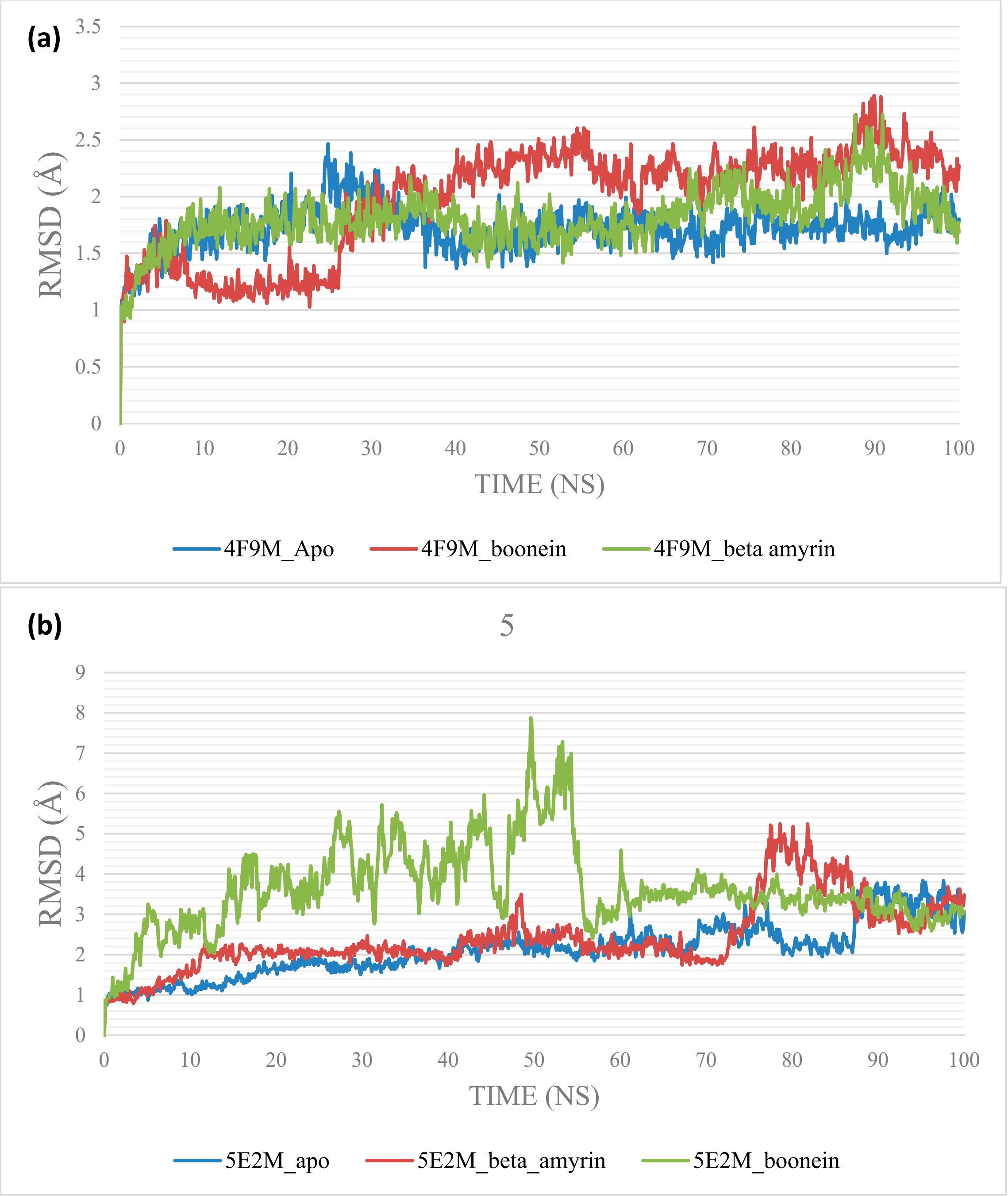

Figure S1. The Backbone-Root Mean Square Deviation (RMSD) plots of molecular dynamics (MD) simulation of ligands complexed to (a) human PPARgamma-LBD (4F9M) (b) human carbonic anhydrase isozyme I (**5E2M)**.

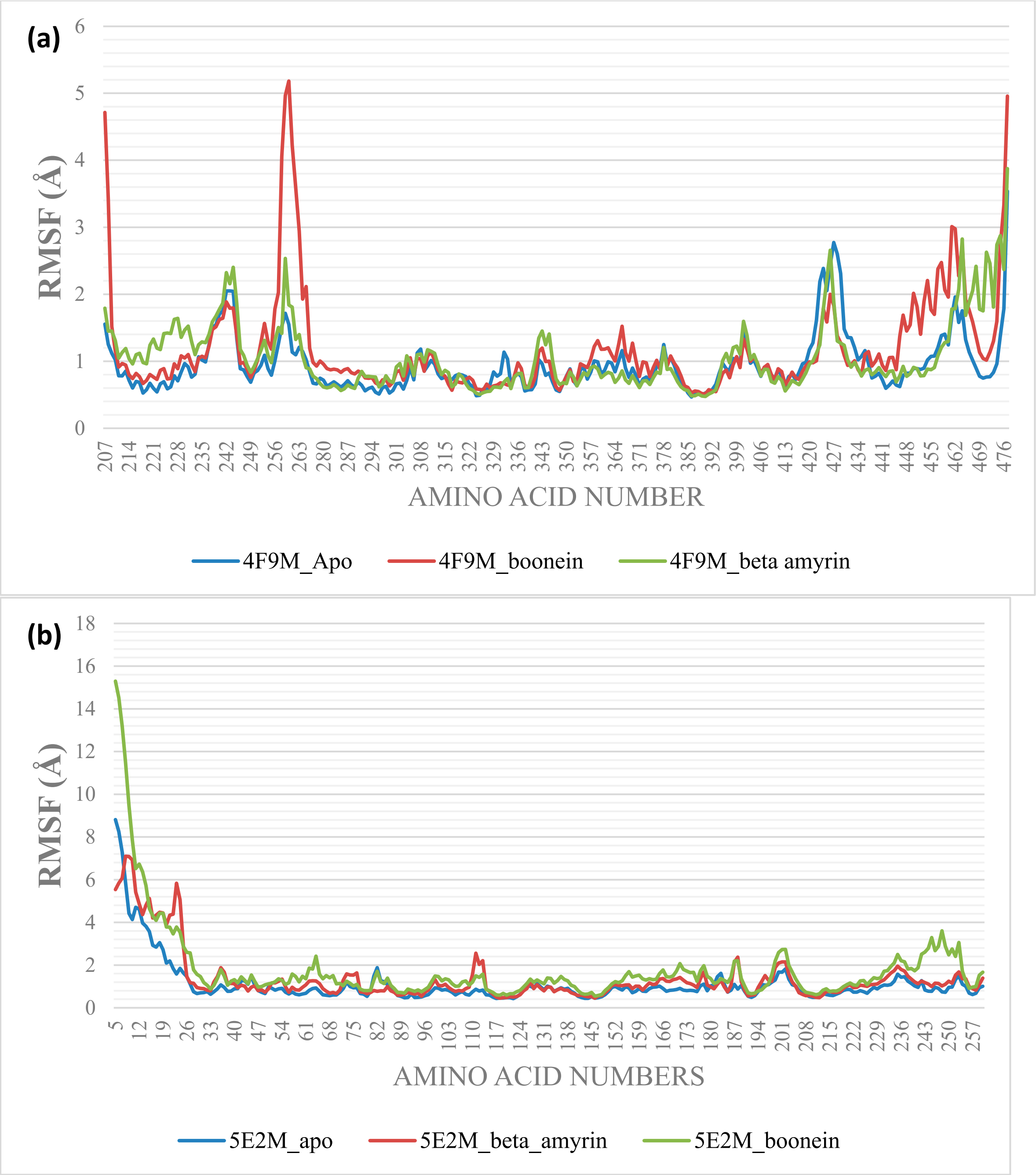

Figure S2: Per residue Root Mean Square Fluctuations (RMSF) plots of molecular dynamics (MD) simulation of ligands complexed to (a) human PPARgamma-LBD (4F9M) (b) human carbonic anhydrase isozyme I (**5E2M)**.

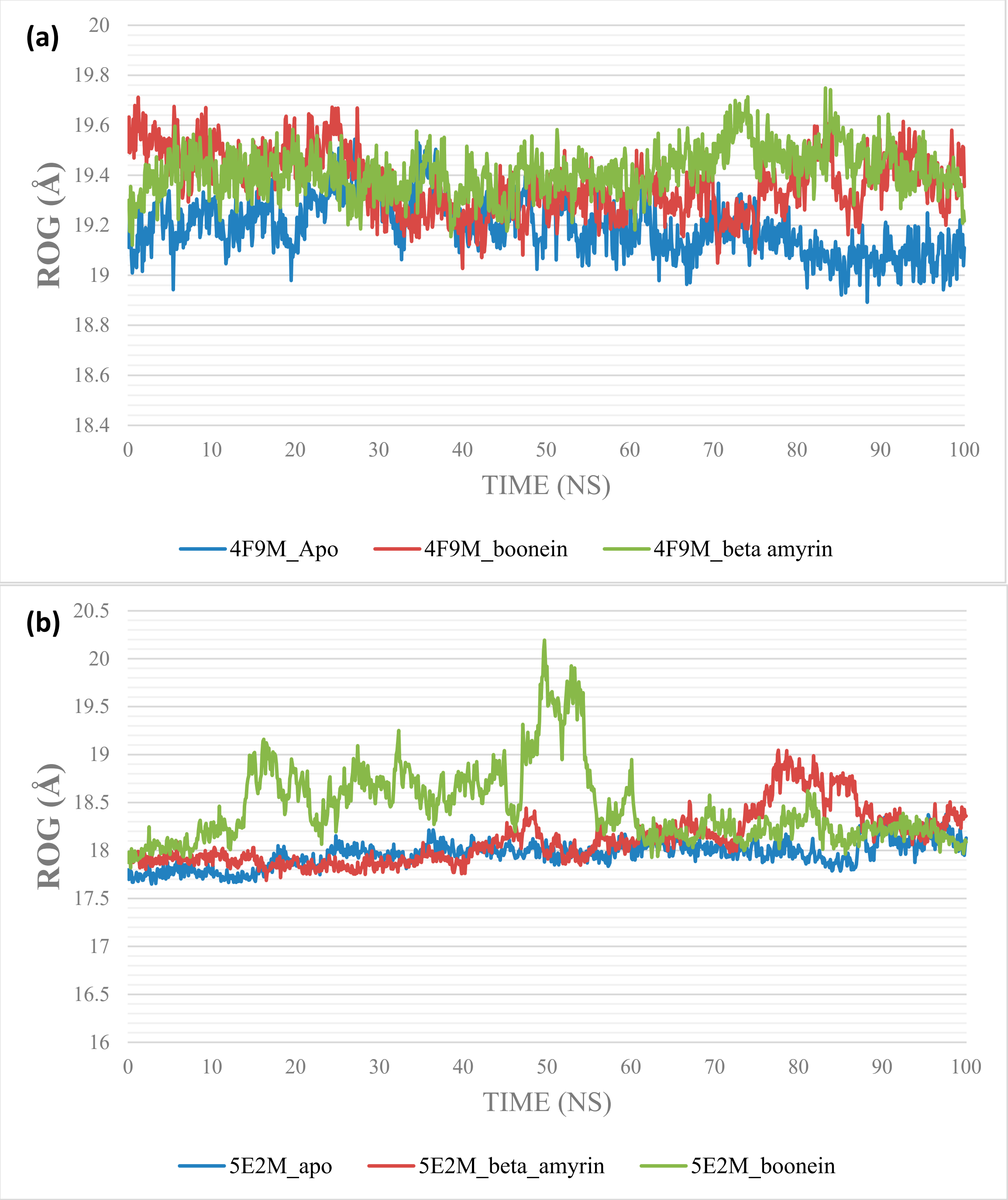

Figure S3: The Radius of gyration (RoG) plots of molecular dynamics (MD) simulation of ligands complexed to (a) human PPARgamma-LBD (4F9M) (b) human carbonic anhydrase isozyme I (**5E2M)**.

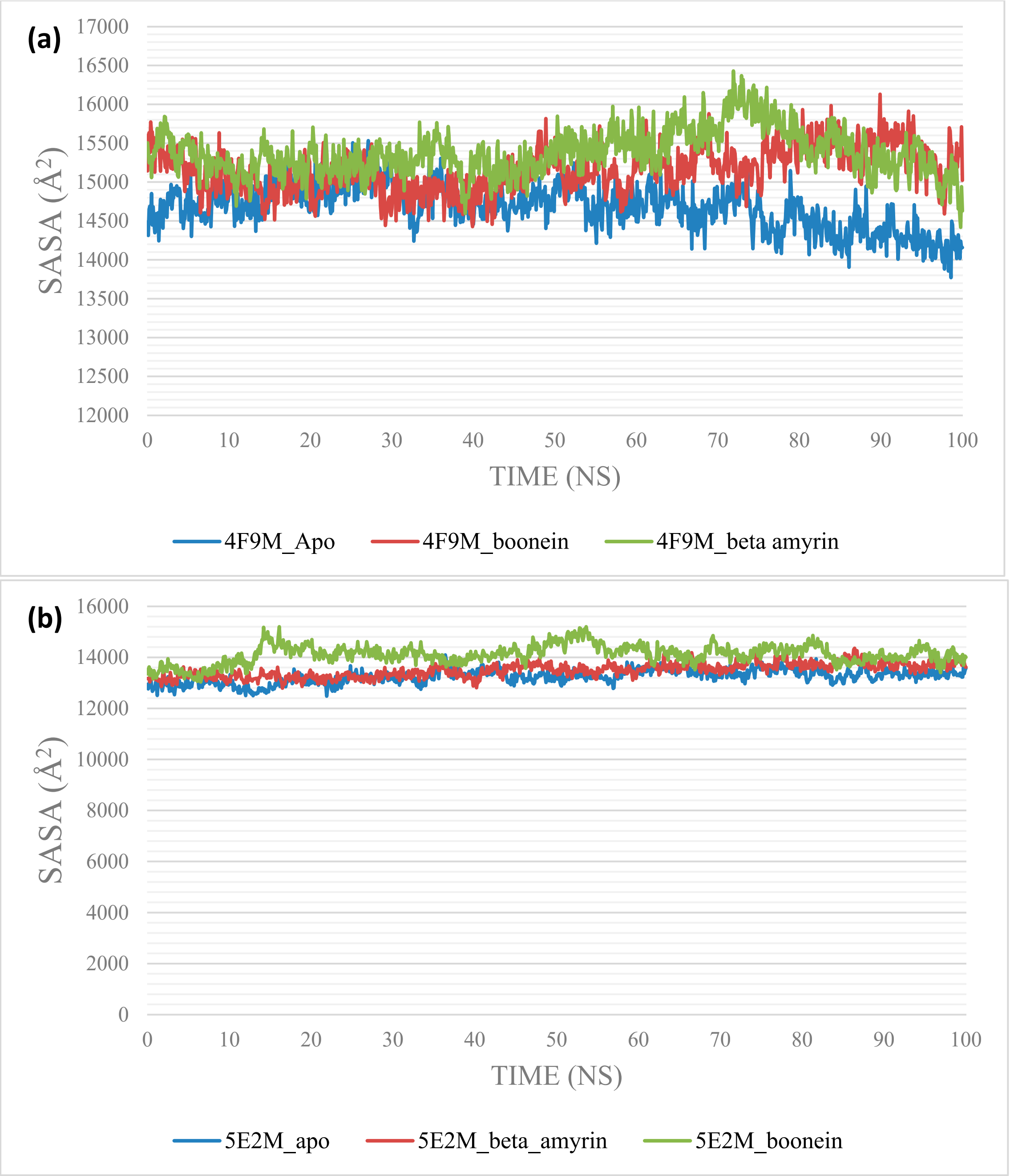

Figure S4: The Surface Accessible Surface Area (SASA) plots of molecular dynamics (MD) simulation of ligands complexed to (a) human PPARgamma-LBD (4F9M) (b) human carbonic anhydrase isozyme I (**5E2M)**.

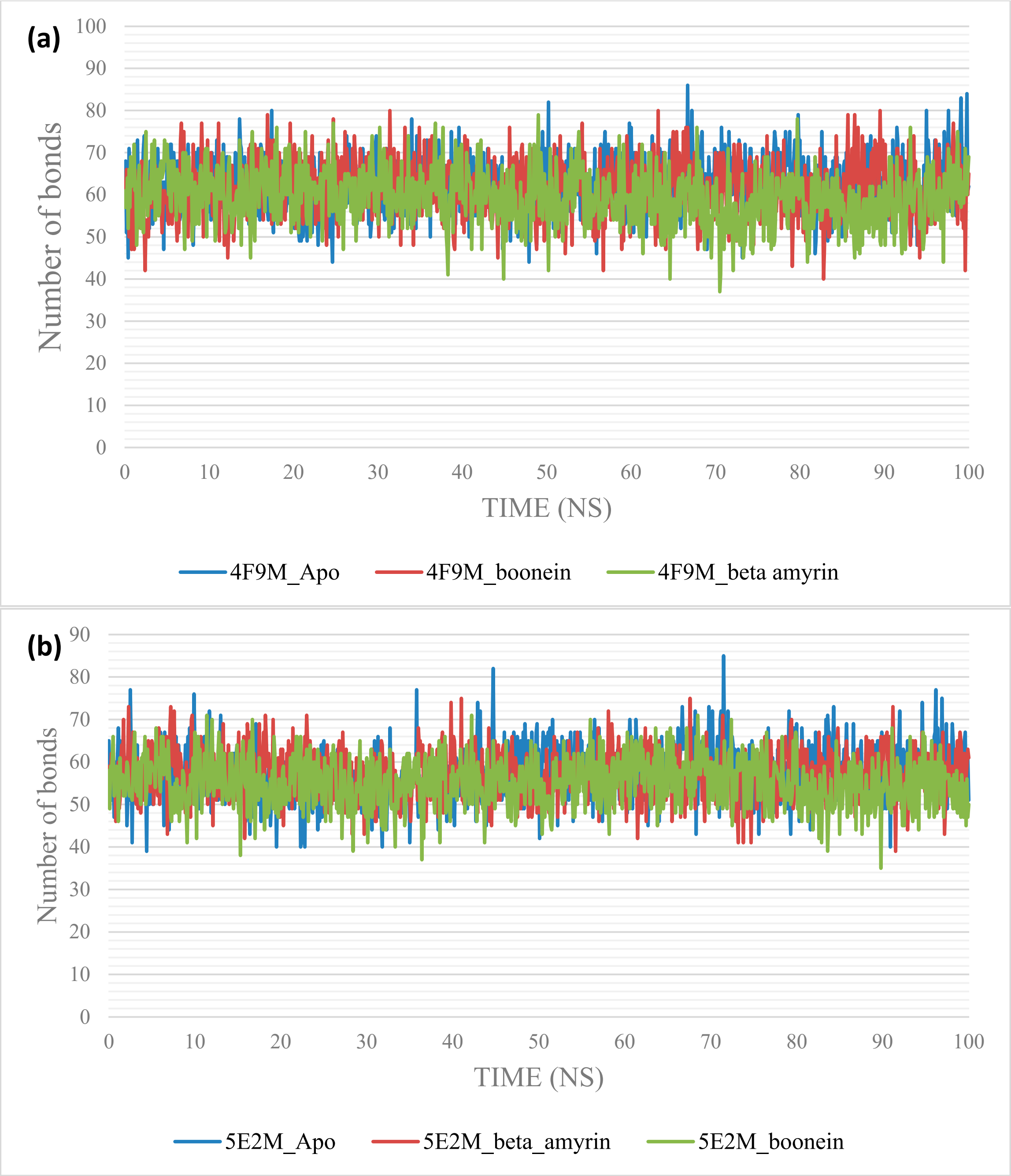

Figure S5: The changes in the number of H-bonds during the MDS trajectory of ligands complexed to (a) human PPARgamma-LBD (4F9M) (b) human carbonic anhydrase isozyme I (**5E2M)**.

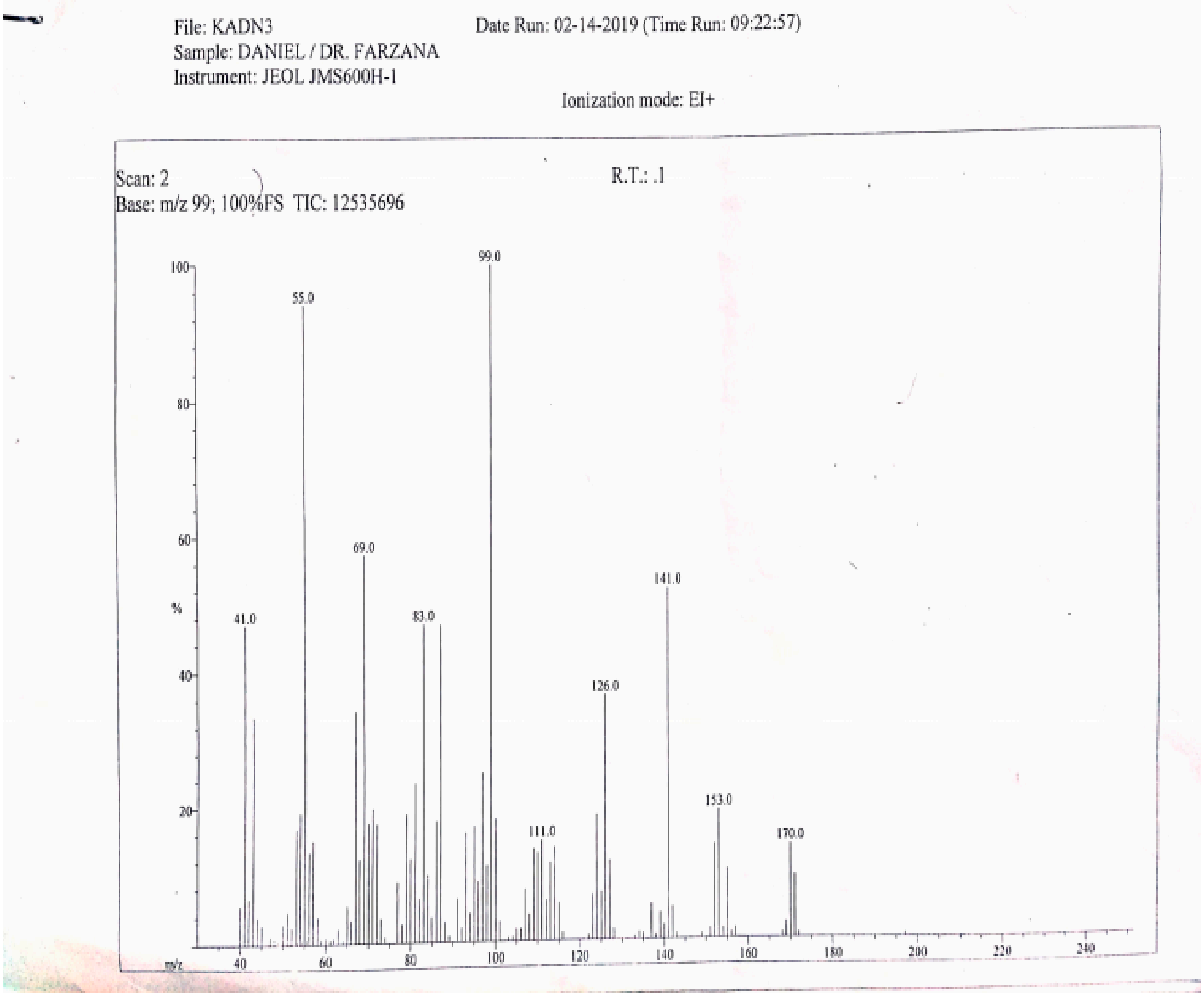

Figure S6: Mass Spectrum of Compound 1 isolated from DCM fraction of *Alstonia boonei* stem-bark.

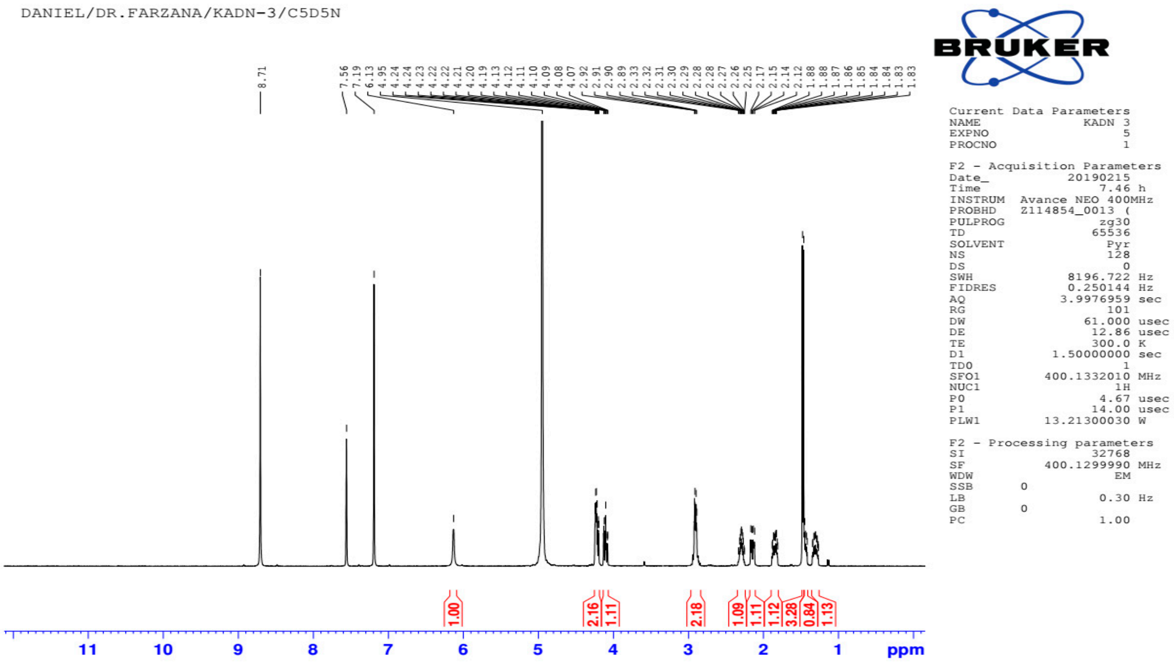

Figure S7: Proton (^{1}H) NMR Spectroscopy of Compound 1 isolated from DCM fraction of *Alstonia boonei* stem-bark.

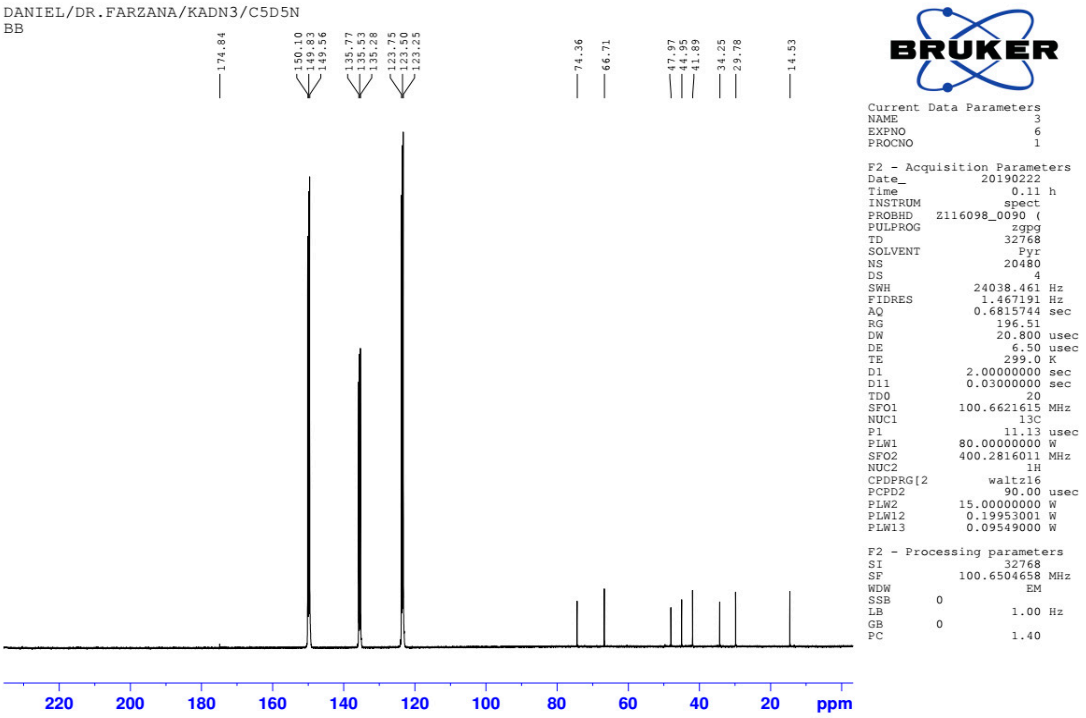

Figure S8: Carbon 13 (^{13}C) NMR Spectroscopy of Compound 1 isolated from DCM fraction of *Alstonia boonei* stem-bark.

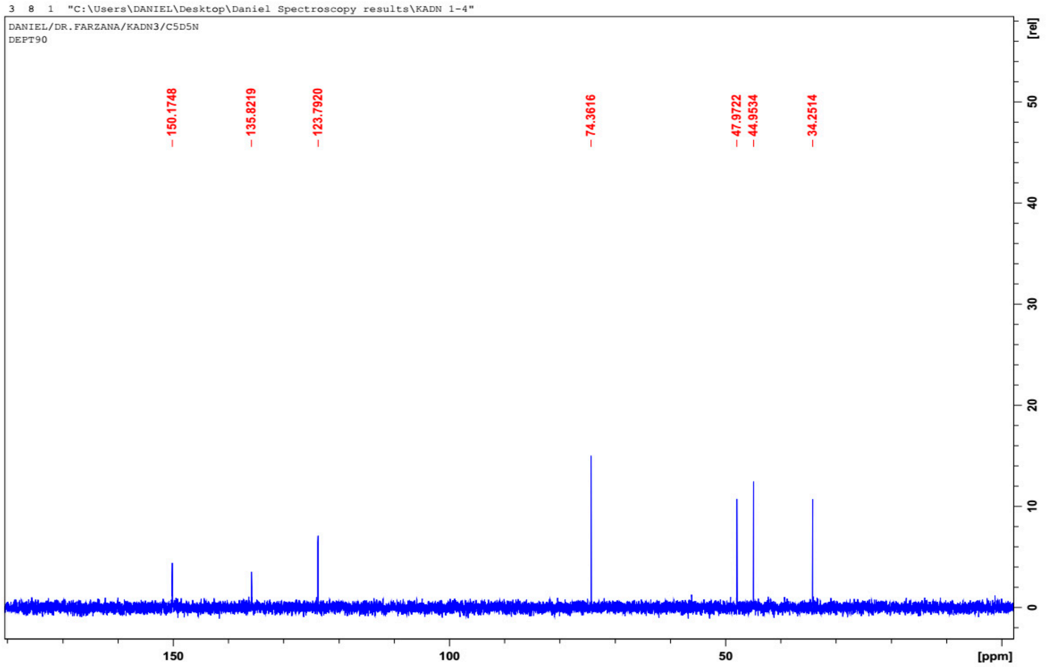

Figure S9: DEPT 90 (Distortionless Enhancement by Polarisation Transfer) Spectrum of Compound 1 isolated from DCM fraction of *Alstonia boonei* stem-bark.

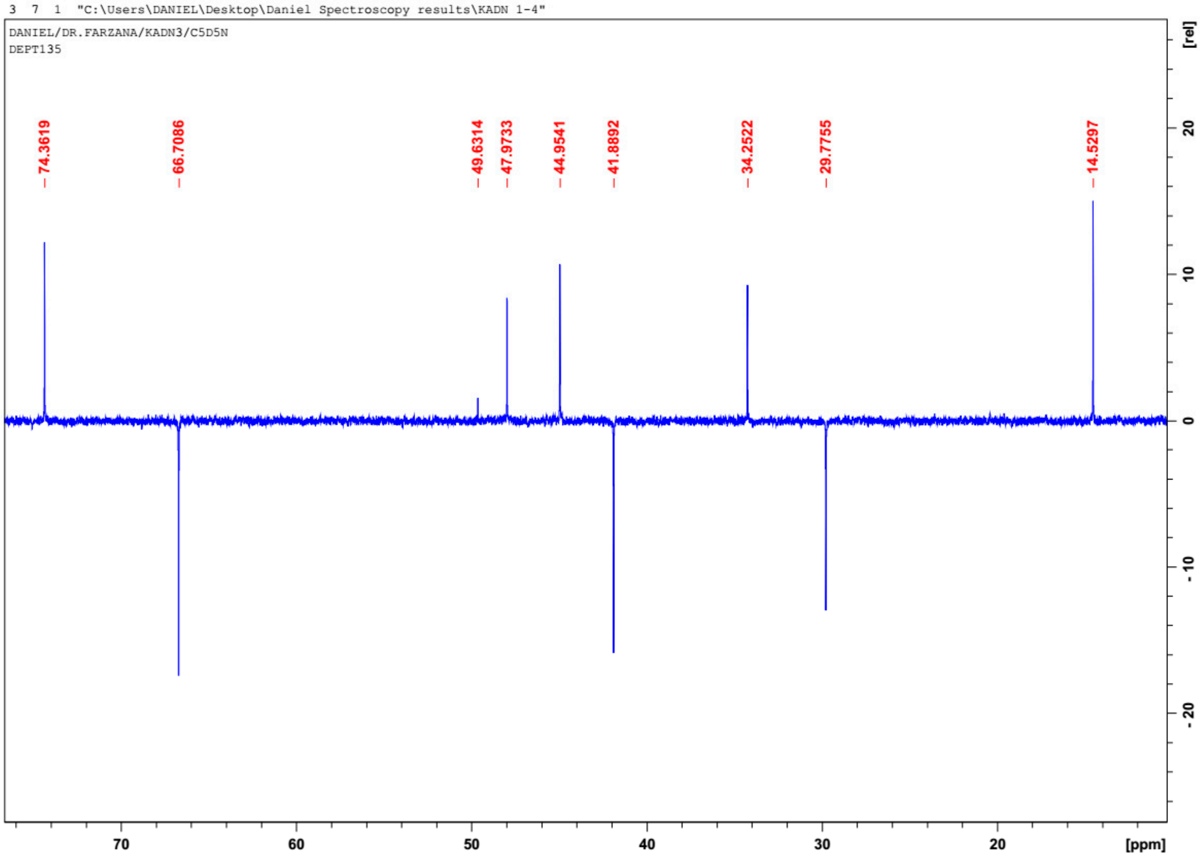

Figure S10: DEPT 135 (Distortionless Enhancement by Polarisation Transfer) Spectrum of Compound 1 isolated from DCM fraction of *Alstonia boonei* stem-bark.

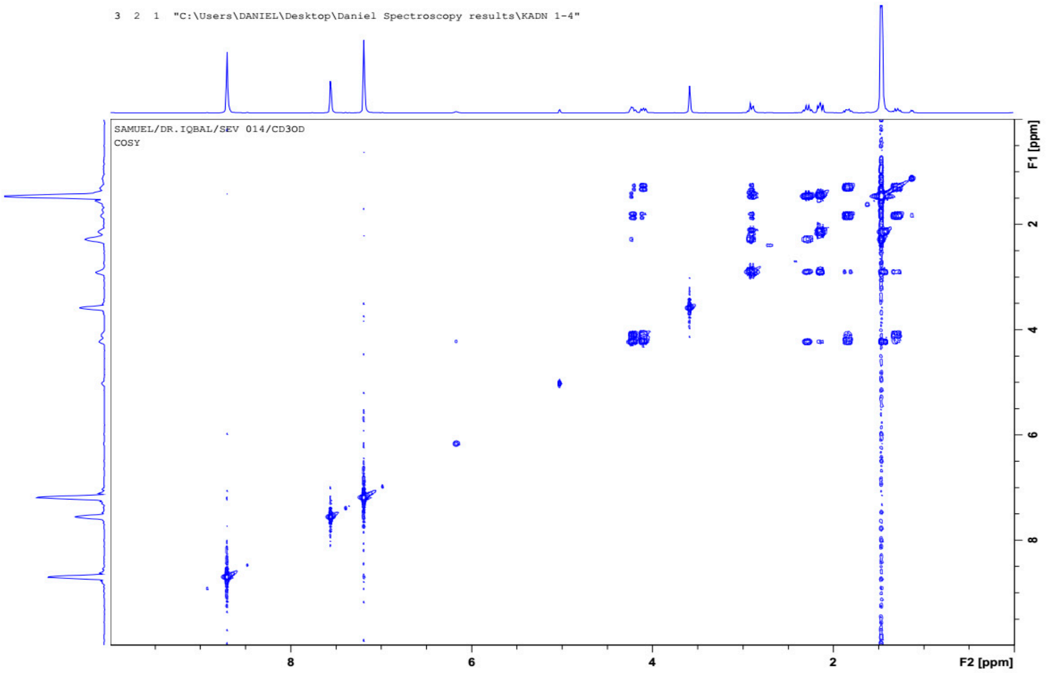

Figure S11: COSY Spectrum of Compound 1 isolated from DCM fraction of *Alstonia boonei* stem-bark.

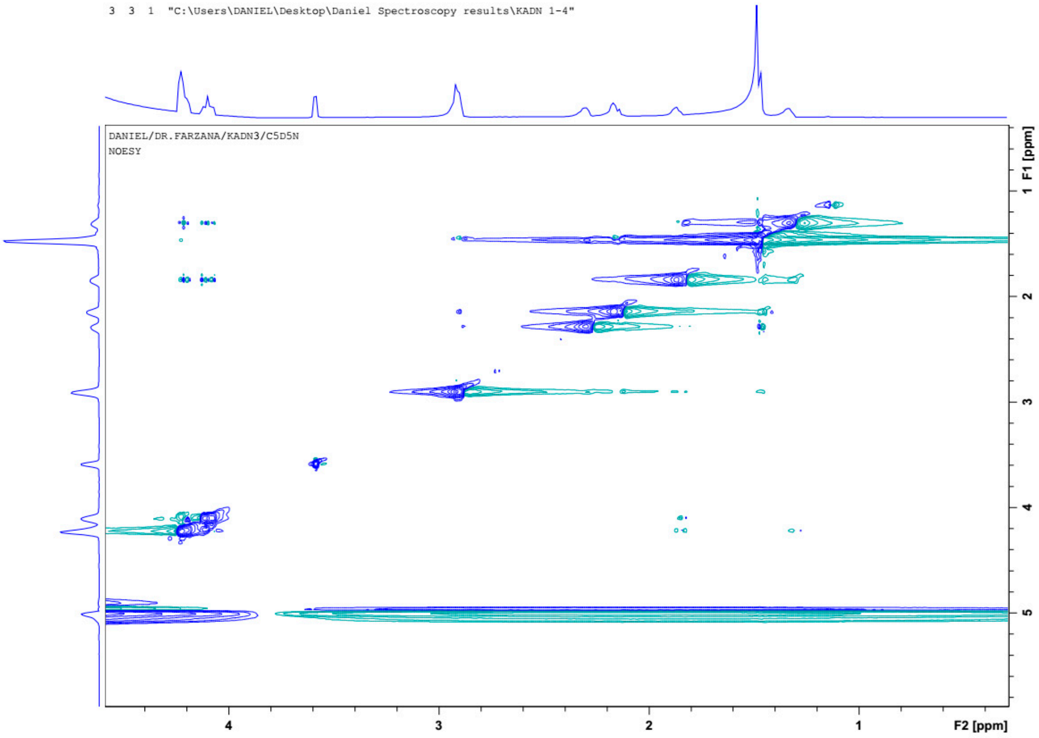

Figure S12: NOESY spectrum of Compound 1 isolated from DCM fraction of *Alstonia boonei* stem-bark.

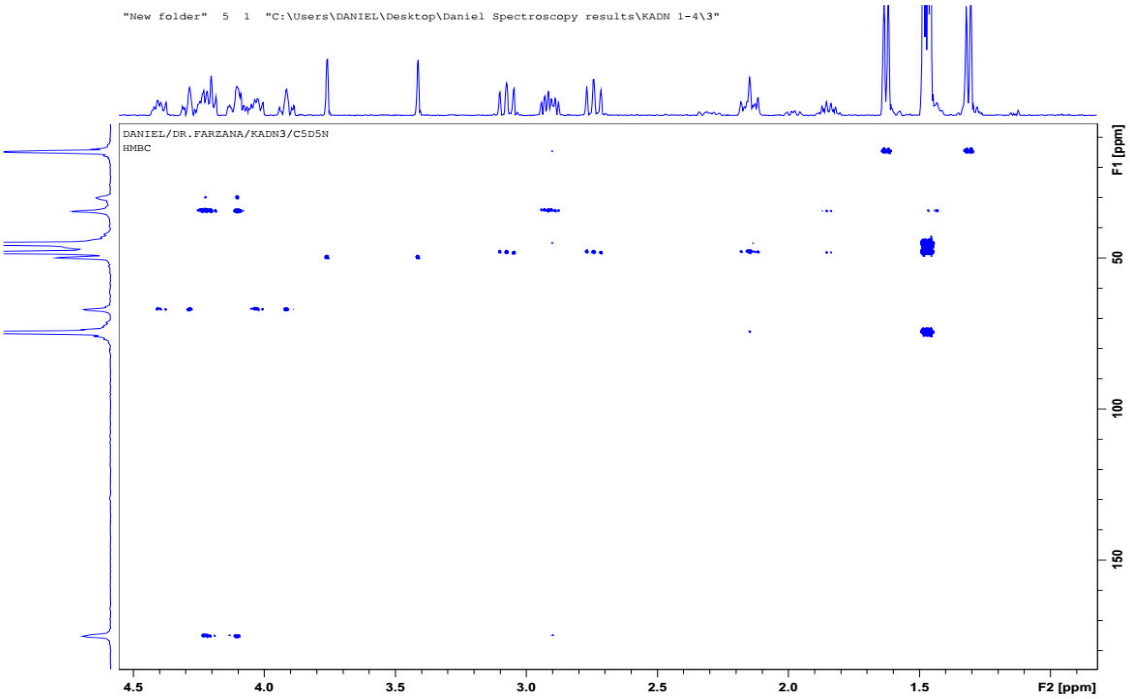

Figure S13: Heteronuclear Multiple Bond Correlation Spectrum of Compound 1 isolated from DCM fraction of *Alstonia boonei* stem-bark.

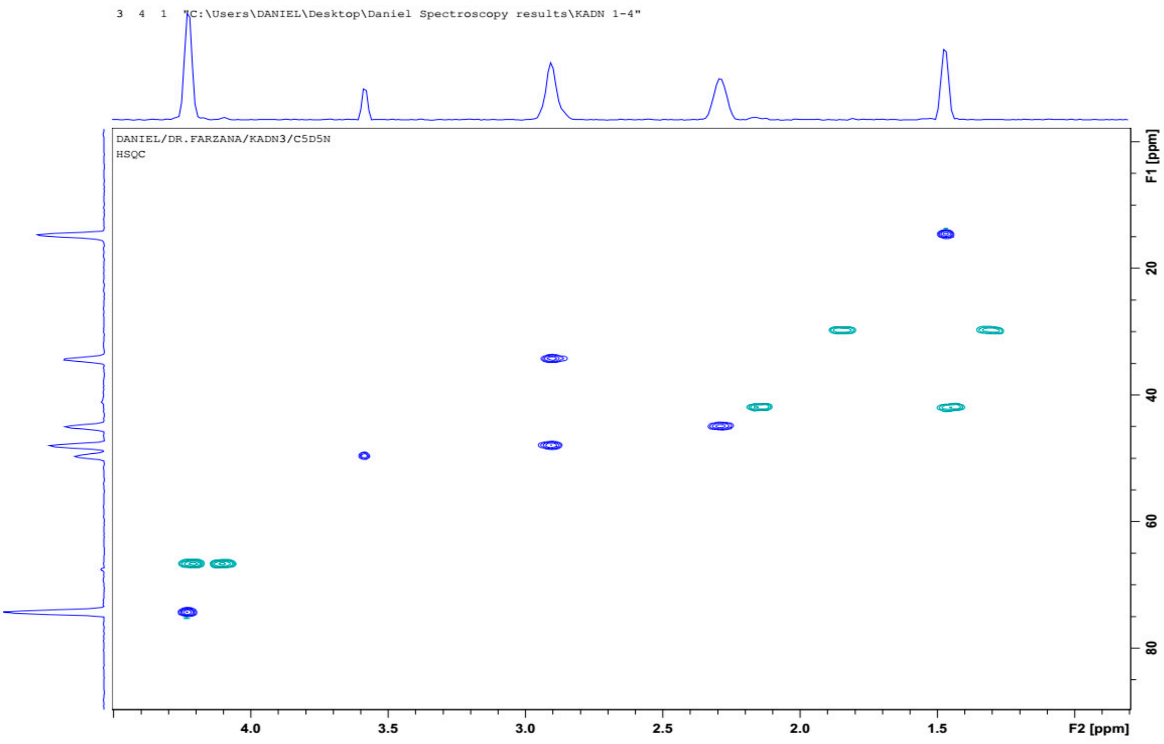

Figure S14: Heteronuclear Single Quantum Coherence spectrum of Compound 1 isolated from DCM fraction of *Alstonia boonei* stem-bark.

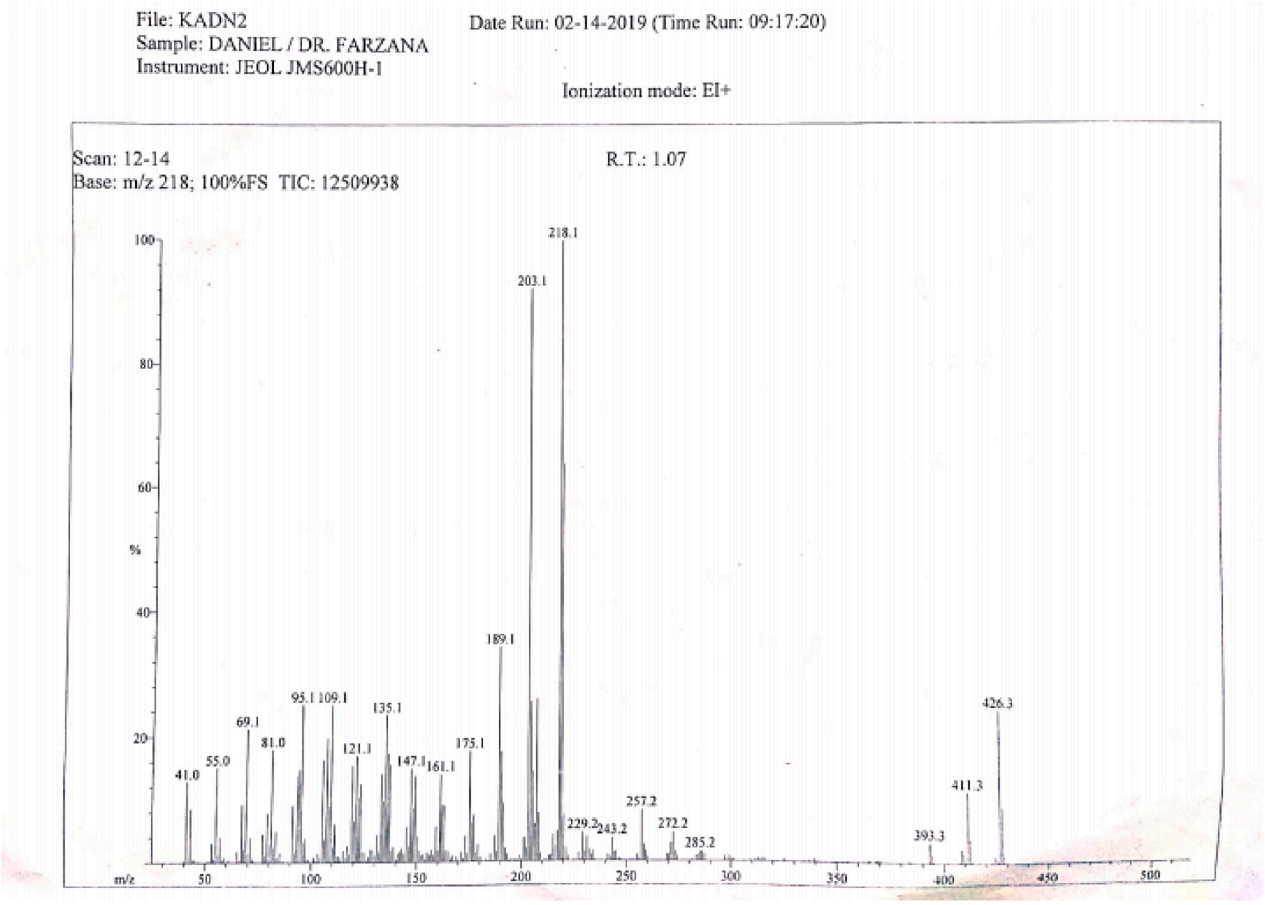

Figure S15: Mass spectrum of Compound 2 isolated from DCM fraction of *Alstonia boonei* stem-bark.

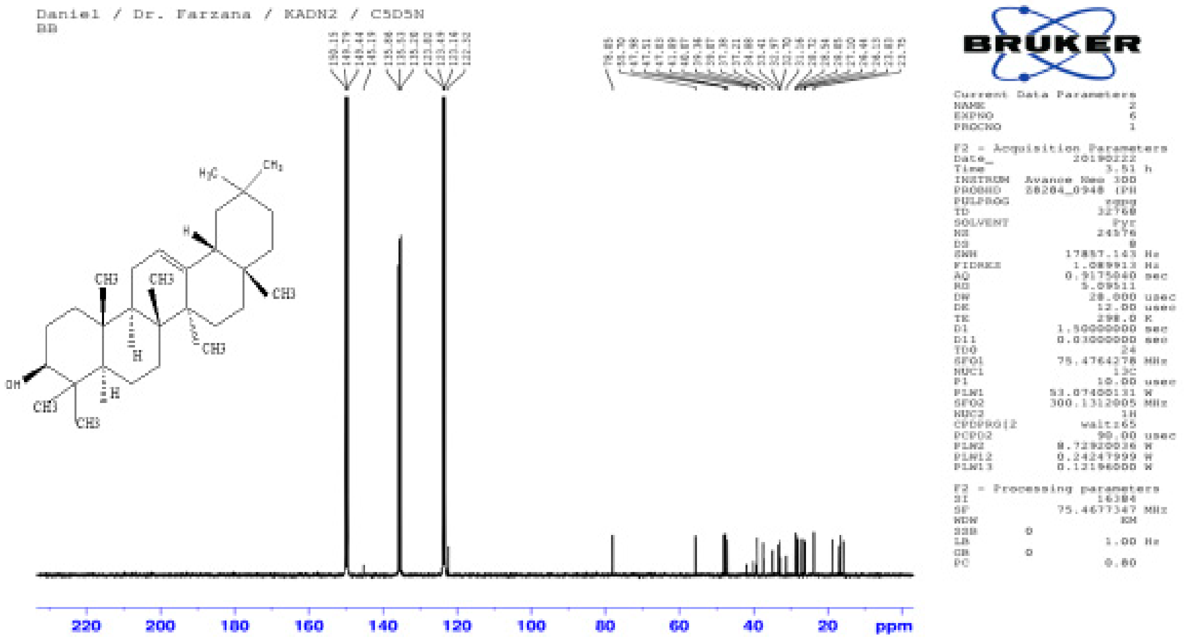

Figure S16: Carbon 13 (^{13}C) NMR Spectroscopy of Compound 2 isolated from DCM fraction of *Alstonia boonei* stem-bark.

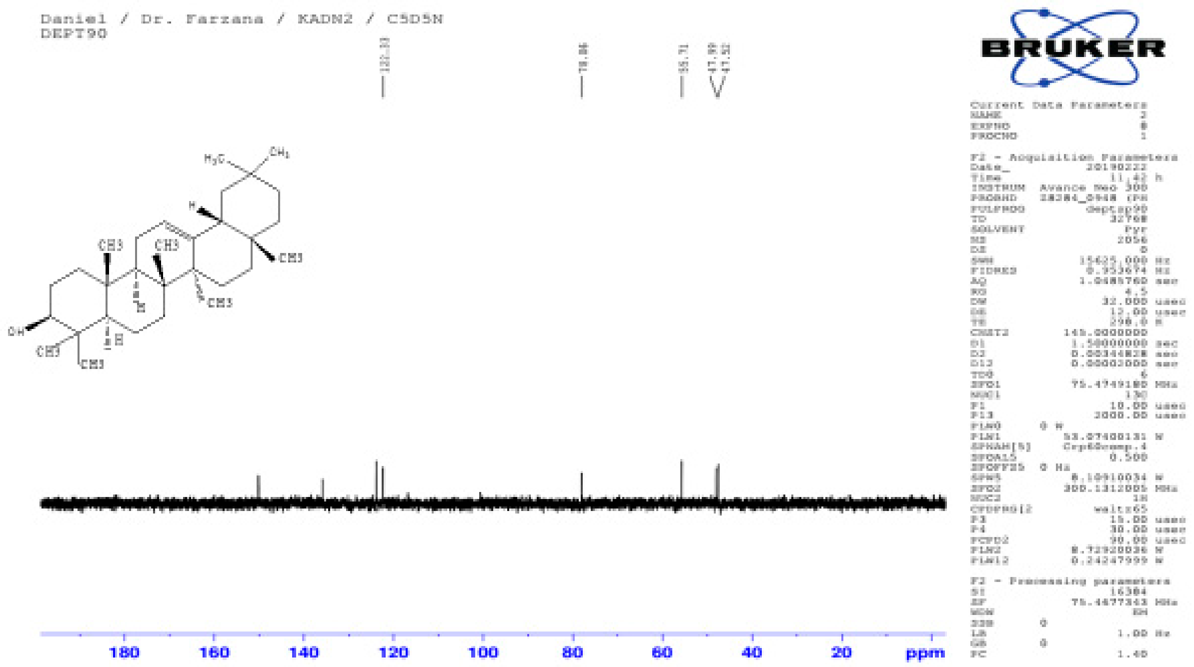

Figure S17: DEPT 90 (Distortionless Enhancement by Polarisation Transfer) Spectrum of Compound 2 isolated from DCM fraction of *Alstonia boonei* stem-bark.

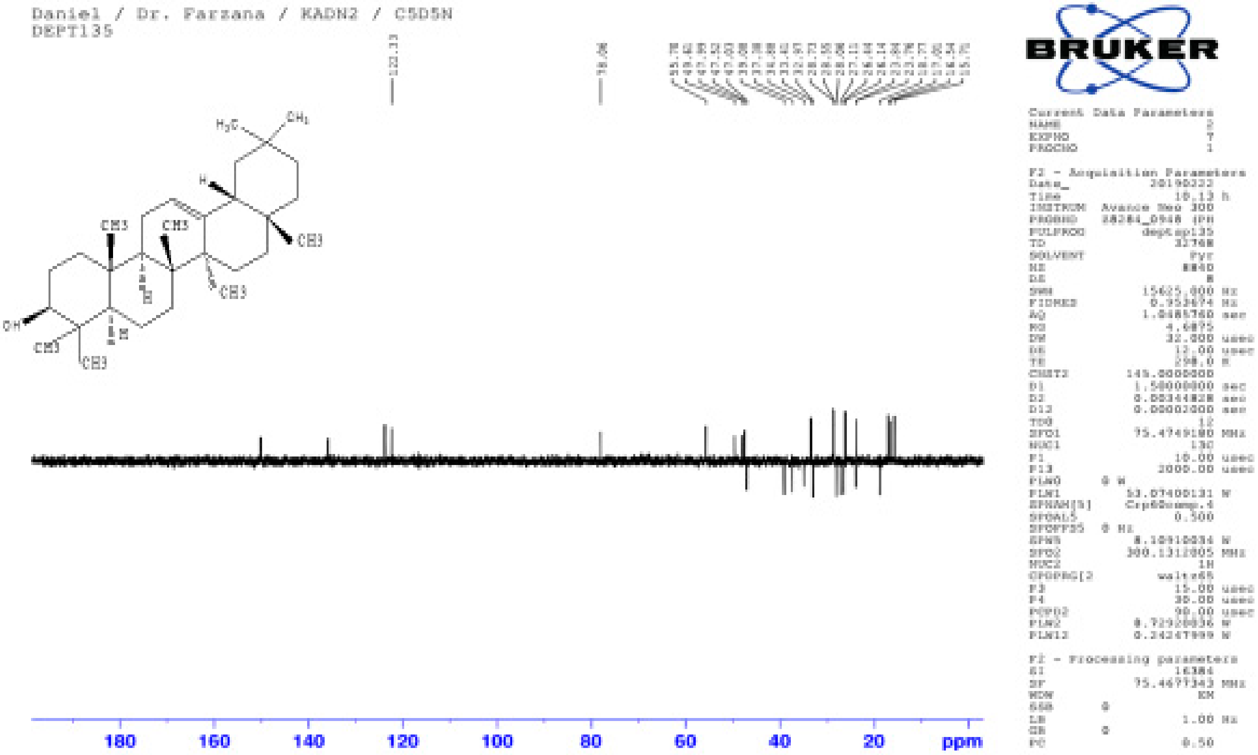

Figure S18: DEPT 135 (Distortionless Enhancement by Polarisation Transfer) Spectrum of Compound 2 isolated from DCM fraction of *Alstonia boonei* stem-bark.

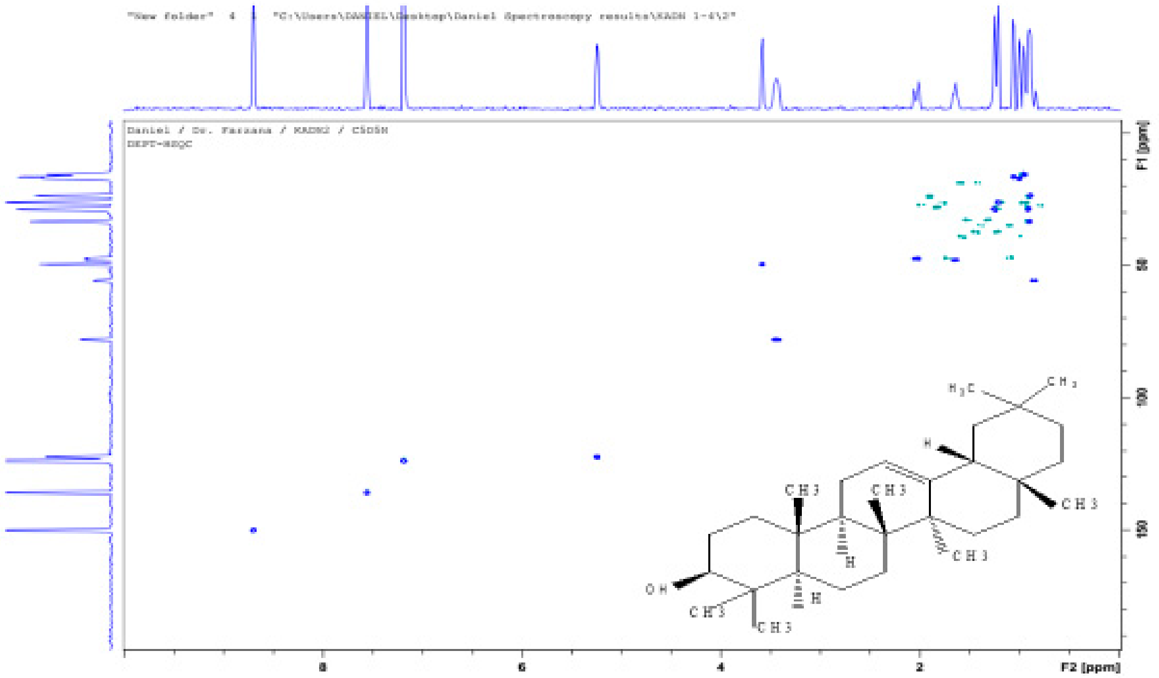

Figure S19: Heteronuclear Single Quantum Coherence Spectroscopy of Compound 2.

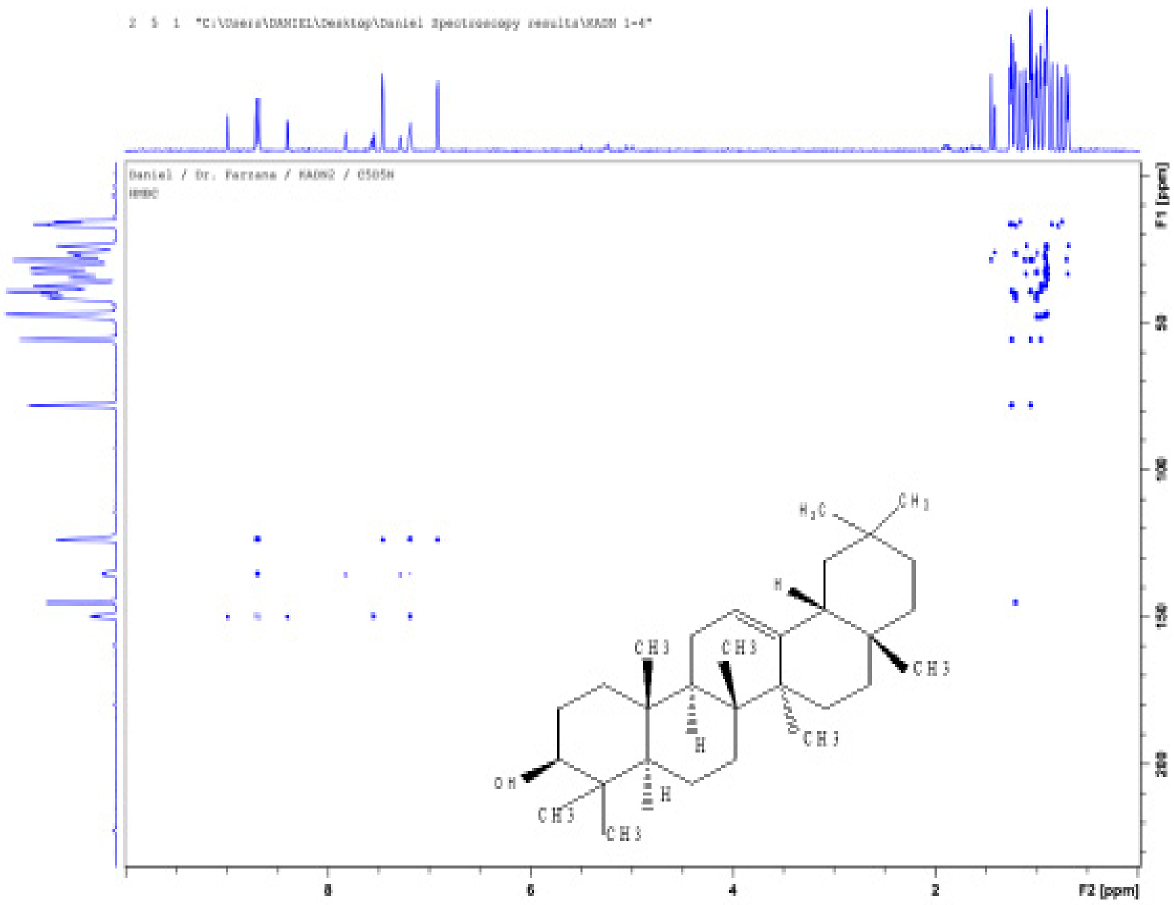

Figure S20: HMBC Spectroscopy of Compound 2 isolated from DCM fraction of *Alstonia boonei* stem-bark.
